# Parent-Child Mutual Influences on Sugar-Sweetened Beverage Consumption Behaviors: Actor-Partner Analysis

**DOI:** 10.2196/76943

**Published:** 2025-07-24

**Authors:** May O Lwin, Allison Seet, Seraphina Leo, Peter J Schulz

**Affiliations:** 1 Wee Kim Wee School of Communication and Information Nanyang Technological University Singapore Singapore; 2 Institute of Communication and Health Università della Svizzera italiana Lugano Switzerland

**Keywords:** Parent-child influence, parental mediation, healthy behavior, sugar-sweetened beverages, Singapore health promotion

## Abstract

**Background:**

Childhood obesity remains a significant global public health issue, with consumption of sugar-sweetened beverages (SSBs) recognized in scientific studies as a key contributing factor. While family influences on children’s dietary behaviors and their effects on obesity risk are well-documented, the dynamics between parents and children in shaping SSB consumption remain underexplored.

**Objective:**

Drawing on social influence theory, this study examines how parent-child perceptions and consumption intentions regarding SSBs are interrelated and the potential mediating role of attitudes. We also studied how engagement with food-related content on social media may relate to consumption intentions.

**Methods:**

We conducted a face-to-face survey of 250 parent-child dyads (N=500) living in public housing in Singapore, a country tackling overweight in its population. Dichotomous items were used to measure cognitive perceptions and attitudes toward SSBs. Both parent and child participants self-reported their intention to consume SSBs. Based on the World Health Organization (WHO) definition of SSBs, visuals of culturally relevant drink products were used in the survey to visualize SSBs. Dyadic data analysis using the MEDYAD tool was conducted to test the actor-partner interdependence model (APIM) and examine the impact of the parent-child dyadic relationship on individuals’ intention to consume sugary drinks.

**Results:**

Pearson correlation results indicated positive associations between parent and child cognitive perceptions and intentions to consume SSBs. Path analysis revealed strong actor effects with both parents’ (β=.52, *P*<.001) and children’s (β=.43, *P*<.001) own perceptions predicting their attitudes and subsequent intentions to consume SSBs (parent: β=.32, *P*<.001; child: β=.31, *P*<.001). Partner effects also emerged: parental perceptions influenced children’s intentions (β=.20, *P*=.01), while children’s perceptions shaped parental attitudes (β=.20, *P=*.02), highlighting the reciprocal nature of influence. While digital food media engagement was positively associated with consumption intentions, its effects were relatively modest and not central to the dyadic pathways.

**Conclusions:**

Our study findings highlight the reciprocal effects of both parents and children in influencing healthier behaviors and hence provide insights to aid obesity prevention efforts. By addressing the interdependent associations of parent-child dynamics, this research bridges theory and health communication practice, offering a novel framework for combating obesity through family-centered approaches.

## Introduction

### Background

The rising prevalence of childhood obesity has become a significant public health issue, with rates among Singapore’s school-aged children (aged 6-18 years) increasing from 11% in 2013 to 16% in 2021 [1]. This rapid upward trend is evident even among primary 1 (grade 1) students, the youngest entrants into Singapore’s school system, where national data from the Singapore Department of Statistics indicate a steady increase in the proportion of overweight and severely overweight children in this age group—from 9.8% in 2014 to 12.1% in 2022 [2]. As childhood obesity becomes more prevalent, it is no longer solely a pediatric issue, but a pressing public health crisis at the national level.

Among the various dietary contributors, the frequent consumption of sugar-sweetened beverages (SSBs) has been consistently identified as a major risk factor for childhood obesity, with growing scientific evidence linking high sugar intake to adverse weight outcomes in early childhood [3-5]. This concern is similarly echoed in national dietary data showing that Singaporean adults consume an average of 60 grams of sugar daily—exceeding the recommended intake by 10 grams—with more than half of this sugar derived from SSBs [6]. Excessive sugar consumption, particularly through SSBs, has been associated with increased caloric consumption, greater adiposity, and a higher risk of developing noncommunicable and metabolic diseases such as type 2 diabetes, hypertension, and nonalcoholic fatty liver disease [7-9]. With these dietary patterns, unsurprisingly, Singapore has reported one of the highest rates of diabetes among developed countries [10]. The implications are far-reaching—from higher hospitalization rates to reduced work productivity and an increased risk of developing other chronic diseases [11]—and place a burden on public health resources.

Globally, the intake of SSBs among children and adolescents is also rising, increasing alarmingly by 23% over the past 3 decades [12]; this trend is raising concerns over the impact of SSB consumption on nutritional quality and future health outcomes. Frequent consumption of SSBs during childhood is associated with elevated blood pressure, dental caries, and unhealthy dietary habits, factors that, as discussed earlier, can contribute to the early onset of noncommunicable and metabolic diseases such as obesity and type 2 diabetes. Moreover, these patterns often persist into adulthood [12,13], potentially reinforcing a life-course trajectory of poor health outcomes. In Singapore, the widespread popularity of sugar-laden drinks—such as the highly popular bubble tea (often sweetened with high-fructose corn syrup), carbonated soft drinks, and sweetened fruit juices—coupled with pervasive advertising of SSBs has contributed substantially to the growing burden of childhood obesity [14]. Children and adolescents today are immersed in social media environments saturated with food-related content, often encountered passively through algorithm-driven feeds, influencer promotions, and peer sharing. Evidence suggests that such incidental exposure can subtly shape adolescents’ cravings, food preferences, and consumption behaviors over time, including increased inclination toward energy-dense, nutrient-poor products such as SSBs [5,15]. Considering this, reducing SSB consumption through key socializing agents is critical for obesity prevention and health promotion strategies targeted at younger populations.

Children’s dietary behaviors are not formed in isolation but rather through complex social interactions embedded within their daily environments. While social influence theory highlights the role of multiple agents (eg, peers, teachers, and the media), the family unit is considered one of the earliest and most influential socializing agents in shaping children’s health behaviors [16-20]. Parent-child interactions related to broader dietary norms and health-related behaviors serve as a foundation through which children begin to interpret, model, and internalize parental attitudes, beliefs, and practices about food, nutrition, and health [21]. As primary socialization agents, parents play a critical role, often acting as gatekeepers of the home food environment and influencing children’s eating patterns and dietary choices from an early age; in this way, parents provide guidance and set expectations for healthy consumption. As such, parental attitudes and behaviors toward SSBs can directly affect children’s consumption patterns.

Previous studies have investigated the links between parental modeling and children’s food consumption, as well as between media exposure and dietary intake in parent-child dyads [18,22]. However, most existing research has focused on unidirectional influences from parents to children, with comparatively less attention given to bidirectional or reciprocal effects such as how children’s own preferences, attitudes, or media exposure might influence parental perspectives and behaviors surrounding SSB consumption. Moreover, studies that systematically examine the bidirectional dynamics in parent-child dyads specifically in the context of SSB consumption are lacking. In particular, the psychological mechanisms that drive and sustain these dynamics, such as the role of cognitive perceptions and positive attitudes toward SSBs, are still unclear.

Emerging perspectives informed by social influence theory challenge the notion that children are passive recipients of parental influence [23], instead highlighting their active role in shaping and responding to social cues related to food and health behaviors. This includes influencing household norms and practices through reciprocal interactions with their parents. Building on Kelman’s foundational work [23], contemporary social influence research has expanded to encompass a broader set of cognitive mechanisms, including normative-informational influence, central-peripheral information processing, and unconscious or automatic pathways [24-26]. These developments underscore the multifaceted nature of social influence, which operates at both conscious and subconscious levels, shaping children’s dietary behaviors across diverse interpersonal contexts.

There is a clear gap in knowledge regarding the reciprocal influence between parents and children in understanding parents’ and children’s dietary behaviors in the current age of digital media. In today’s media-saturated environment, children and adolescents’ food preferences and consumption habits are increasingly shaped—and often reinforced—by food-related content on social media. As children mature and become more independent, their opinions and preferences can contribute to household decision-making, including food-related choices such as grocery selections and meal planning. Such exposure not only influences their own individual consumption patterns, but can also indirectly influence parental attitudes, grocery purchasing decisions, and potentially shift household dietary norms. In the Singaporean context, where educational milestones are closely tied with distinct social development stages, children’s evolving roles within the family—across primary, secondary, and post-secondary levels—may reflect varying degrees of influence over family dietary behaviors and food-related decision-making, and vice versa.

### Objectives

**Figure 1:**
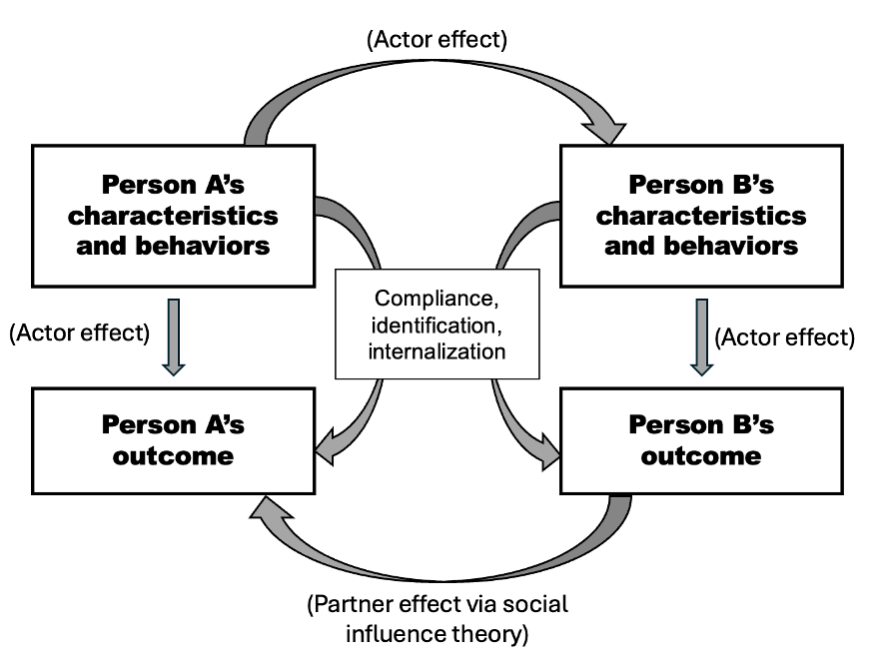
Conceptual framework depicting potential actor-partner effects in dyadic relationships using the actor-partner interdependence model.

To examine the reciprocal nature of influence within parent-child actor-partner relationships, this research draws upon the actor-partner interdependence model (APIM), situated within the broader theoretical lens of social influence theory. APIM allows us to disentangle the extent to which an individual’s perceptions affect their own behavior (*actor effects*), as well as how those perceptions influence the behavior of the other dyad member (*partner effects*). In the context of SSB consumption, this framework enables a more granular understanding of how attitudes, perceptions, and intentions for SSB purchase are shaped by parent-child relationships across different stages of social development. Figure 1 presents a self-developed conceptual framework illustrating potential actor-partner effects in dyadic relationships using APIM [27,28].

The overarching aim of this study is to examine how SSB-related attitudes, perceptions, and behavior intentions are associated within parent-child dyads in Singapore, exploring patterns of mutual influence using a cross-sectional dyadic framework. It further examines how the influence of the parent-child dynamic on SSB consumption intent varies across children’s developmental stages and social media exposure. This research is thus guided by the following overarching research questions: (1) how do parents’ and children’s perceptions of SSBs influence each other’s attitudes and behavioral intentions, and (2) how do these effects vary across developmental stages and digital media exposure levels.

### Hypotheses

To address the research objectives, the following hypotheses were proposed based on social influence theory:

#### Hypothesis 1

Hypothesis 1 (H1) is related to actor effects via intrapersonal pathways:

- H1a (parent actor effect) is that parents’ perceptions of SSBs relate to their behavioral intentions to consume these beverages, with this relationship mediated by their attitudes toward SSBs.
- H1b (child actor effect) is that children’s perceptions of SSBs relate to their own behavioral intentions to consume these beverages, with this relationship mediated by their attitudes toward SSBs.

#### Hypothesis 2

Hypothesis 2 (H2) is related to partner effects via interpersonal pathways:

- H2a (parent-to-child partner effect) is that parents’ perceptions of SSBs are expected to predict their children’s behavioral intentions to consume these beverages, with this relationship mediated by the children’s attitudes toward SSBs.
- H2b (child-to-parent partner effect) is that children’s perceptions of SSBs are expected to predict their parents’ behavioral intentions to consume these beverages, with this relationship mediated by the parents’ attitudes toward SSBs.

#### Hypothesis 3

Hypothesis 3 (H3) is related to moderators of partner effects:

- H3a (moderation by developmental stage) is that the child-to-parent partner effect is expected to be stronger for older children than younger children.
- H3b (moderation by social media exposure) is that the child-to-parent partner effect is expected to be stronger for children with higher levels of social media exposure.

## Methods

### Study Design and Population

The cross-sectional, in-person survey was conducted in Singapore, targeting parent-child dyads. Participants were recruited using a multistage cluster sampling procedure based on a compiled list of government primary and secondary schools, stratified by region according to the Singapore Urban Redevelopment Authority’s zoning. This approach ensured geographic diversity and representation across urban planning districts in Singapore. The targeted child age range (8-19 years) corresponds to key developmental stages from late childhood through adolescence, during which independent dietary preferences begin to form and parental influence remains critical.

Within each region of the country, schools were randomly selected. The public housing estates feeding the selected schools were then identified, and data collection began from the top floor of the lowest-numbered apartment block adjacent to each school, proceeding door-to-door. From there, participants were recruited through snowball sampling. Eligibility criteria included (1) being a Singaporean citizen or having permanent resident status, (2) parents and children residing in the same household, and (3) children being aged between 8 and 19 years. Each household contributed one parent-child dyad. In families with more than one eligible child in the targeted age range, the participating parent was given the discretion to select one child to take part in the study. While this approach may introduce some selection bias, no additional controls were applied for within-household selection, consistent with protocols in comparable dyadic studies prioritizing feasibility in field-based recruitment.

Both parents and children completed parallel versions of the survey assessing their perceptions, attitudes, and intentions regarding SSB consumption. The survey was administered in person in English. Survey items were designed to be age appropriate, with simple and clear language used throughout to accommodate younger participants. Prior to data collection, the research team conducted internal pilot testing and cognitive walk-throughs to ensure item clarity and relevance. Where necessary, terminology was simplified or rephrased to enhance understanding. For younger participants, researchers were present to guide them through the survey to ensure they could complete it independently, thereby minimizing parental influence on the children’s responses.

As part of the survey, both parents and children were shown 4 unbranded images of commonly consumed beverages in Singapore: a glass of cola, bubble tea, a sports drink, and a bottle of orange juice (Multimedia Appendix 1). These images were presented simultaneously at the start of the relevant survey section and served as visual references for participants when responding to questions on cognitive perceptions and attitudes toward SSBs. The images were selected based on the WHO definition of SSBs and adapted for cultural relevance [29].

### Ethical Considerations

Informed consent was obtained from both parents and children prior to participation. Participants were informed that they could withdraw from the study at any point during data collection without any consequences, and that doing so would not affect their relationship with the university. The study protocol was approved by the University’s Institutional Review Board (IRB-2022-320) and data collection took place between 2023 and 2024. No personal identifiers were collected. Instead, each dyad was assigned a unique identification code used during data analysis to maintain confidentiality. Upon completion of both the parent and child surveys, each household received a $40 supermarket voucher.

### Survey Measures

The measurement of parent-child dyads’ perceptions and attitudes toward SSBs was adapted from previous validated studies [30-32]. Perceptions toward SSBs were measured using 4 dichotomous items on a 5-point semantic differential scale (“unhealthy” to “healthy,” “unsafe” to “safe,” “unnatural” to “natural”). One item with low factor loading was excluded from the analysis. Internal consistency for the final 3-item scale was acceptable, with the McDonald ω values exceeding the commonly recommended threshold of 0.70 (ω=0.71 for parents, ω=0.76 for children). In comparable samples of children aged 9-12 years, Lwin et al [31] reported high reliability for a similar 4-item scale assessing views on unhealthy foods, including sugary drinks (Cronbach α=0.93).

Positive attitudes toward SSBs were measured with four 5-point semantic differential items (“not at all happy” to “very happy,” “not at all satisfied” to “very satisfied”). Internal consistency was excellent in the current sample (ω=0.96 for parents; ω=0.94 for children). In another comparable study by Lwin et al [32] involving 210 parent-child dyads (children aged 9-15 years), a similar attitude scale toward healthy food yielded Cronbach α=0.87.

Intention to consume SSBs was measured using a single item, adapted from previously validated instruments [32], asking participants to rate their agreement with the statement “I intend to drink these beverages in the next week” on a 5-point Likert scale from “strongly disagree” to “strongly agree.” This item had previously demonstrated predictive validity in studies of adolescent dietary behavior.

In addition, children’s exposure to food-related content on social media was measured by their self-reported frequency of seeking such content, using a 5-point Likert scale ranging from “strongly disagree” to “strongly agree.”

Demographic data were collected from both parents and children, including age, sex, and ethnicity. Children’s school grade levels were used to classify them into three social age groups reflective of the Singapore education system: (1) ages 8-12 years, (2) ages 13-16 years, and (3) ages 17-19 years. This categorization captures key educational transitions in Singapore and was used as a covariate in regression analyses to account for developmental differences in autonomy, social influence, and decision-making relevant to dietary behaviors.

### Statistical Analysis

Data analyses were conducted using SPSS Statistics (version 29; IBM Corporation) along with the MEDYAD tool developed by Coutts et al [33]. Descriptive statistics were computed to summarize participant demographics. Pearson correlation coefficients were calculated to explore bivariate associations among key study variables.

To examine the dyadic associations between perceptions, attitudes, and consumption intentions, the APIM was applied using path analysis with 1000 bootstrap samples [33,34]. Standardized regression coefficients (β) were reported for all effects. A 2-tailed significance level of *P*<.05 was used to determine statistical significance.

## Results

### Sample Characteristics

A total of 250 parent-child dyads participated in the study, totaling 500 respondents (Table 1). The average age of parent participants was 43.7 (SD 5.77) years, with 82% (205/250) being female and 86.8% (217/250) being of Chinese ethnicity. Among child participants, the mean age was 12.1 (SD 3.52) years, with almost equal sex distribution (female: 126/250, 50.4%; male: 124/250, 49.6%). The majority of children were Chinese (219/250, 87.6%), followed by Indian (18/250, 7.2%), Malay (8/250, 3.2%), and other ethnicities (including Burmese, Eurasian, Filipino, and Indonesian; 5/250, 2%).

Children’s grade levels, classified by social age groups, showed that 141 of 250 (56.4%) were in primary school (grades 1-6), 77 of 250 (30.8%) were in secondary school (grades 7-10), and 32 of 250 (12.8%) were in post-secondary school (grades 11 and 12).

**Table 1:** Demographic information of study participants.

Characteristics			Parents (n=250)		Children (n=250)
Age (years), mean (SD)			43.7 (5.77)		12.1 (3.52)
**Sex, n (%)**					
	Female	205 (82)		126 (50.4)	
	Male	45 (18)		124 (49.6)	
**Ethnicity, n (%)**					
	Chinese	217 (86.8)		219 (87.6)	
	Malay	8 (3.2)		8 (3.2)	
	Indian	18 (7.2)		18 (7.2)	
	Other^a^	7 (2.8)		5 (2.0)	
**Grade, n (%)**					
	1-6	—^b^		141 (56.4)	
	7-10	—		77 (30.8)	
	11-12	—		32 (12.8)	

^a^Other ethnicities included Burmese (n=1), Eurasian (n=1), Filipino (n=2), Indonesian (n=2), and Russian (n=1) for parents and Burmese (n=1), Eurasian (n=2), Filipino (n=1), and Indonesian (n=1) for children.

^b^Not applicable.

### Correlations Between Variables

Table 2 summarizes Pearson correlation coefficients between key study variables. Notably, parents’ cognitive perceptions of SSBs were positively associated with their positive attitudes toward (*r*=0.35, *P*<.001) and intentions to consume (*r*=0.21, *P*<.001) SSBs. A similar pattern was observed among children: children’s perceptions were positively correlated with their attitudes (*r*=0.36, *P*<.001) and intentions (*r*=0.14, *P*=.03).

**Table 2:** Correlations between study variables (n=250).

Variable		Parents’ perception toward SSBs^a^	Parents’ positive attitudes toward SSBs	Parents’ intention to consume SSBs	Children’s perception toward SSBs	Children’s positive attitudes toward SSBs	Children’s intention to consume SSBs	Children’s social age group	Children’s food media exposure
**Parents’ perception toward SSBs **									
	*r*	1	0.354^b^	0.214^b^	0.151^c^	0.026	0.102	0.033	0.009
	*P* value	—^d^	<.001	<.001	.02	.68	.11	.61	.89
**Parents’ positive attitudes toward SSBs **									
	*r*	0.354^b^	1	0.353^b^	0.151^c^	0.220^b^	0.206^b^	–0.257^b^	0.039
	*P* value	<.001	—	<.001	.02	<.001	.001	<.001	.54
**Parents’ intention to consume SSBs **									
	*r*	0.214^b^	0.353^b^	1	0.127^c^	0.068	0.325^b^	–0.081	0.184^b^
	*P* value	<.001	<.001	—	.045	.29	<.001	.21	.004
**Children’s perception toward SSBs **									
	*r*	0.151^c^	0.151^c^	0.127^c^	1	0.363^b^	0.136^c^	0.154^c^	0.073
	*P* value	.02	.02	.045	—	<.001	.03	.02	.25
**Children’s positive attitudes toward SSBs **									
	*r*	0.026	0.220^b^	0.068	0.363^b^	1	0.275^b^	0.056	0.061
	*P* value	.68	<.001	.29	<.001	—	<.001	.39	.34
**Children’s intention to consume SSBs **									
	*r*	0.102	0.206^b^	0.325^b^	0.136^c^	0.275^b^	1	0.081	0.169^b^
	*P* value	.11	.001	<.001	.03	<.001	—	.21	.008
**Children’s social age group **									
	*r*	0.033	–0.257^b^	–0.081	0.154^c^	0.056	0.081	1	0.167^b^
	*P* value	.61	<.001	.21	.02	.39	.21	—	.01
**Children’s food media exposure**									
	*r*	0.009	0.039	0.184^b^	0.073	0.061	0.169^b^	0.167^b^	1
	*P* value	.89	.54	.004	.25	.34	.008	.01	—

^a^SSBs: sugar-sweetened beverages.

^b^The correlation is significant at the *P<*.01 level (2-tailed).

^c^The correlation is significant at the *P<*.05 level (2-tailed).

^d^Not applicable.

Our overarching research objective questioned the links between parents’ and children’s perceptions of SSBs and their influences on each other’s attitudes and behavioral intentions. The data revealed that significant associations between parents and children emerged in their perceptions and behaviors. Parents’ and children’s perceptions of SSBs were weak but still significantly correlated (*r*=0.15, *P*=.02), while their consumption intentions showed moderate correlation (*r*=0.33, *P*<.001). Children’s social age group showed a positive correlation with their perceptions of SSBs (*r*=0.15, *P*=.02), suggesting that older children held more favorable views of these beverages. Interestingly, social age was negatively correlated with parents’ positive attitudes toward SSBs (*r*= –0.26, *P*<.001), indicating potential shifts in parental attitudes as children mature. Although children’s food-related media exposure was positively correlated with both their parents’ (*r*=0.18, *P*=.004) and their own (*r*=0.17, *P*=.008) intentions to consume SSBs, the effect sizes were small. While these findings indicate some individual-level associations, the food media exposure variable was not directly tied to the dyadic framework central to the APIM. To preserve the theoretical integrity and parsimony of the APIM, which is designed to capture reciprocal interpersonal effects within dyads, children’s food-related media exposure was excluded from the final model, as its role as an individual-level predictor was not conceptually aligned with the model’s core assumptions. Hence, H3b, which aimed to assess the influence of social media exposure, was excluded from further analysis.

### Path Analysis (APIM) and Regression Analysis

**Figure 2:**
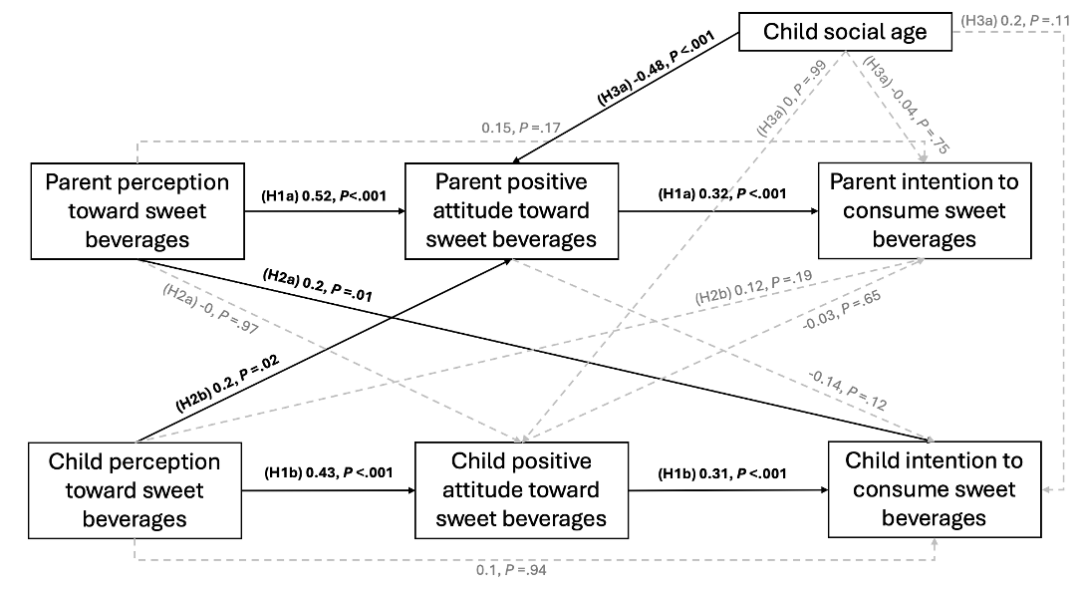
Path analysis results using the actor-partner interdependence model. Standardized regression coefficients and P values are reported. H1: hypothesis 1; H2: hypothesis 2; H3: hypothesis 3.

To examine H1a (parent actor effect), H1b (child actor effect), H2a (parent-to-child partner effect), and H2b (child-to-parent partner effect), we conducted path analysis of our APIM model. Additional regression analysis was further conducted to the test moderation effects of H3a (moderation by developmental stage). The results are summarized in Figure 2.

H1 proposed actor effects via intrapersonal pathways for both parents (H1a) and children (H1b). Actor effects demonstrated that both parents’ and children’s cognitive perceptions strongly predicted their own positive attitudes toward SSBs (parent: β=.52, *P*<.001; child: β=.43, *P*<.001). In turn, these positive attitudes were significantly associated with higher intentions to consume SSBs (parent: β=.32, *P*<.001; child: β=.31, *P*<.001).

H2 proposed partner effects via interpersonal pathways, with H2a predicting a parent-to-child partner effect and H2b predicting a child-to-parent partner effect. Data analysis showed that partner effects further illuminated patterns of mutual associations between parents and children. Specifically, parents’ cognitive perceptions significantly predicted their children’s intentions to consume SSBs (β=.20, *P*=.01). Notably, children’s perceptions also positively influenced their parents’ attitudes toward SSBs (β=.20, *P*=.02), highlighting the reciprocal nature of influence within the dyad. Overall, the model supports the hypothesized pathways of H1 and H2, with both actor and partner effects influencing SSB consumption intentions in parent-child dyads.

To further explore how parents’ and children’s perceptions relate to consumption intentions, we ran multiple regression analyses, as shown in Table 3. Initially, we included both parents’ income and education level as covariates to control for socioeconomic status. However, neither variable was statistically significant (income: β=–.008, *P*=.52; education level: β=–.062, *P*=.12), and their inclusion did not meaningfully change the results. Therefore, these covariates were excluded from the final model.

When it came to predicting the intention of parents to consume SSBs, both their own perceptions (β=.214, *P*<.001) and their child’s perceptions (β=.127, *P*=.05) were significant predictors. Like findings from the APIM analysis, this suggests that parents who view SSBs in a more positive light, and whose children also hold favorable views, are more likely to intend to consume such beverages. In contrast, when predicting children’s intention to consume SSBs, only the child’s own perception was a significant predictor (β=.136, *P*=.03), while the parent’s perception was not statistically significant (β=.102, *P*=.11).

**Table 3:** Regression analysis.

			B		SE		β		*t *(*df*)		*P*
**Dependent variable: parents’ intention to consume SSBs **											
	Parents’ perception of SSBs	.341		.099		.214		3.45 (245)		<.001	
	Children’s perception of SSBs	.179		.089		.127		2.01 (245)		.045	
**Dependent variable: children’s intention to consume SSBs **											
	Parents’ perception of SSBs	.175		.109		.102		1.61 (245)		.11	
	Children’s perception of SSBs	.208		.096		.136		2.16 (245)		.03	

## Discussion

### Principal Findings

This study set out to examine the interdependent associations between parents and children in shaping intentions to consume SSBs, drawing on social influence theory and using APIM. Our findings confirmed significant actor and partner effects (H1 and H2), underscoring the reciprocal nature of influence within parent-child dyads. Notably, children’s cognitive perceptions of SSBs significantly influenced their parents’ attitudes, providing novel insight into the evolving role of children as active contributors to family health behaviors.

While active engagement with food-related content on social media was positively associated with both parent and child SSB consumption intentions, these effects were relatively small and outside the primary scope of our dyadic model (proposed H3b). To preserve the conceptual integrity of the APIM, which is designed to assess reciprocal interpersonal influence, this variable was excluded from the final model. Instead, our focus remained on mutual influences within the parent-child relationship, where actor and partner effects provide a more meaningful framework for understanding shared dietary behavior.

To our knowledge, this is the first study to apply APIM to investigate the reciprocal pathways underlying SSB consumption within parent-child dyads. By simultaneously modeling actor and partner effects, APIM offers a more nuanced understanding of intrapersonal and interpersonal processes than traditional unidirectional models. When integrated with social influence theory, this approach was able to capture both the mechanisms of behavioral persuasion and the dynamic flow of influence between family members. This is especially relevant in contemporary applications of the theory, which now recognizes the roles of normative versus informational influence, central versus peripheral processing, and unconscious social mimicry. These mechanisms are especially salient in family contexts, where both overt communication and subtle social cues co-shape health behaviors.

Our findings reaffirm established evidence that individual cognitive perceptions strongly predict positive attitudes and behavioral intentions (H1a and H1b were supported), consistent with health behavior models such as the theory of planned behavior [22,35-37]. These effects underscore the enduring role of individual beliefs in shaping dietary intentions and reinforce the need to address personal cognition in health promotion efforts.

Importantly, our results extend the current literature by quantifying interpersonal dynamics using data from dyadic sources. While previous studies examined the impact of parental influence on children’s dietary choices, they typically relied on unidirectional frameworks, where children are treated as passive recipients of parental modeling [17,38,39]. By contrast, this study demonstrates that children’s perceptions can shape parental attitudes, supporting the idea of upward social influence within the family. This finding offers a statistically grounded contribution to an emerging body of work that recognizes children as socializing agents rather than passive recipients of health messaging.

Parents’ perceptions were positively associated with their children’s intentions to consume SSBs (H2a was supported), a finding consistent with the literature on parental modeling and household food norms [18,40]. More strikingly, children’s positive perceptions also influenced parental attitudes (H2b was supported), suggesting a shift in traditional influence patterns. As children develop preferences and exert greater autonomy, particularly for popular drinks like bubble tea or isotonic beverages, parents may recalibrate their attitudes to accommodate their child’s views, consciously or otherwise [35,36].

These patterns are especially relevant in the context of Singapore, where children’s educational levels correspond with key developmental stages. Our data show that older children tended to hold more favorable views of SSBs, potentially amplifying their influence on household attitudes (H3a was supported). This aligns with social development theory, which posits that adolescents become more persuasive in family decision-making as they gain autonomy [37]. For health promotion and communication practitioners, this suggests that behavior interventions may benefit from tailoring content by age group while recognizing children’s emerging role in shaping household consumption norms.

### Implications

Our findings contribute to the broader application of social influence theory by empirically demonstrating interdependent associations in family health decision-making. While earlier applications of the theory have predominantly emphasized top-down parental influence (parental modeling), more recent expansions have acknowledged reciprocal and automatic forms of influence. By applying APIM, we capture both actor and partner effects within the same analytical framework, offering a more complete understanding of shared health behaviors and extending the application of social influence theory beyond individual-level outcomes.

From a practical perspective, these insights carry implications for obesity prevention strategies. Family-centered interventions that engage both parents and children simultaneously may be more effective than those targeting individuals in isolation. This could include school-based nutrition programs, multigenerational health workshops, or community-based campaigns. Specifically, empowering children with nutrition knowledge and fostering critical thinking around beverage choices could generate spillover effects that reshape parental attitudes and, by extension, household norms. Our findings are also potentially relevant for existing public health policies such as Singapore’s Nutri-Grade labeling system, introduced in 2022 to inform consumers about sugar and saturated fat levels in beverages [41]. While the Nutri-Grade framework targets individual decision-making at the point of purchase, our results suggest that its impact could be magnified through family-based educational efforts. By incorporating parent-child dialogue into nutrition labeling initiatives, health authorities could promote more meaningful engagement with the system and improve long-term dietary behaviors.

### Limitations and Future Research

Some limitations should be considered when interpreting the findings of this study. First, the cross-sectional design limits the ability to draw causal inferences. While the APIM offers a powerful framework for assessing dyadic associations, longer-term observations via longitudinal or experimental designs would allow for a more precise understanding of how the strength and directionality of influence evolve over time, particularly as children mature and gain autonomy and parental control shifts.

Second, the use of self-reported measures may be subject to recall bias or social desirability effects, especially in relation to dietary intentions and perceptions. Future research could benefit from incorporating objective consumption data (eg, food diaries or purchase records) to complement self-reports and enhance measurement accuracy.

Third, although culturally relevant images of SSBs were used to aid recognition, the omission of specific brand names or labels may have influenced participants’ perceptions and engagement with the stimuli. Future studies could experimentally manipulate branding or use ecologically valid consumption scenarios to examine how commercial cues interact with attitudes and intentions.

Additionally, while this study focused specifically on SSBs within parent-child dyads, it is important to consider how other types of familial relationships—such as siblings or grandparents—as well as peer networks may shape children’s dietary behaviors. These wider social influences warrant further exploration in future dyadic or network-based analyses.

Furthermore, our exclusive focus on SSBs represents only one aspect of children’s dietary patterns linked to obesity. Other food categories associated with excess calorie intake and poor nutrition, such as fast food or ultraprocessed snacks, were not examined but may be equally or more salient in shaping health risks. Future research could extend the dyadic framework to examine how parent-child interactions influence consumption of these foods and how such patterns contribute to broader dietary and obesity-related outcomes.

### Conclusion

Despite its limitations, this study strongly advances the current understanding of the reciprocal dynamics between parents and children regarding SSB consumption intentions. By identifying both actor and partner effects, the findings underscore children’s active role in shaping parental attitudes and contribute valuable insights for designing more holistic, family-centered health communication strategies. As childhood obesity continues to challenge an already-strained global public health system, leveraging reciprocal family influences offers a meaningful and actionable pathway for promoting sustainable behavior change related to beverage consumption.

With this study’s findings, we aim to extend existing knowledge on family dynamics in dietary behaviors and present practical insights for health communication strategies targeting both parents and children, particularly within the Singaporean context, with potential for adaptation across other health care systems. By applying the APIM to a health communication context, this study contributes to the broader effort to develop family-centered interventions for obesity prevention, focusing on the interplay of perceptions, attitudes, and intentions that shape beverage consumption decisions.

Looking forward, future research could explore how these parent-child dynamics operate across dietary domains beyond SSBs, such as fast-food consumption or snacking behaviors, which are also known contributors to childhood obesity. Additionally, examining diverse family structures and extending dyadic analyses to peer or sibling relationships could offer deeper insight into the broader social networks that influence children’s eating behaviors. As digital media continues to shape youth culture and food choices, future studies could also investigate how digital exposure intersects with family influence over time, particularly through longitudinal designs that capture developmental shifts in autonomy and influence.

## Appendix

Multimedia Appendix 1 Images of sugar-sweetened beverages.

## Abbreviations

APIM: actor-partner interdependence model
H1: hypothesis 1
H2: hypothesis 2
H3: hypothesis 3
SSB: sugar-sweetened beverage
WHO: World Health Organization
