# Supplementary figures and images for: Parent-Child Mutual Influences on Sugar-Sweetened Beverage Consumption Behaviors: Actor-Partner Analysis
Source: JMIR Pediatr Parent. 2025 Jul 24;8:e76943. doi: 10.2196/76943 (PMC12332451; doi:10.2196/76943)

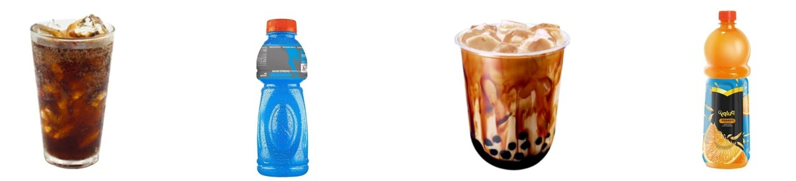

Supplement: Multimedia Appendix 1 [file pediatrics_v8i1e76943_app1.png]
